# Perceived Stress Among Students of Private and Public Sector Medical Colleges of Pakistan: A Cross Sectional Study

**DOI:** 10.1192/j.eurpsy.2022.774

**Published:** 2022-09-01

**Authors:** M. Fatima, Z. Mehdi, S. Saeed, A. Nisar, M. Zain, J. Binte Shakir, I. Aamer, F. Arain, M. Jawad, N. Aziz

**Affiliations:** 1 King Edward Medical University, Psychiatry, Lahore, Pakistan; 2 Nishter Medical University, Psychiatry, Multan, Pakistan; 3 BronxCare Health System Icahn School of Medicine at Mount Sinai, Child & Adolescent Psychiatry, Bronx, United States of America; 4 King Edward Medical University, Department Of Psychiatry, Lahore, Pakistan; 5 Sahiwal Medical College, Physiology, Sahiwal, Pakistan

**Keywords:** Depression, Stress, medical students, psychiatry

## Abstract

**Introduction:**

Medical-education is associated with high overall stress and it is important to identify relevant factors.

**Objectives:**

The study was aimed to discern the differences in perceived stress among the students of public and private medical colleges of Pakistan and to identify factors subservient to any hypothesized difference.

**Methods:**

This cross-sectional study was conducted at different private and public medical colleges of Pakistan using validated tools: PSS-14 (Perceived Stress Scale) to find out the levels of stress faced by each sector and MSSQ (Medical Student Stressor Questionnaire) to determine the factors associated with increased stress.

**Results:**

Total of 424 medical students from various public and private medical colleges of Pakistan (212 each) filled the questionnaires. The mean score +/- SD of PSS-14 was 36.17 ± 6.096 for the public sector and 36.29 ±5.732 for the private sector. Hence, there was no difference between the two comparative means of PSS score, t(422)=-0.213,p=0.831.The results for both sectors were classified as high perceived stress (27-40 score is high perceived stress). Out of 40 individual stress-causing factors in MSSQ, the students from private-sector scored higher as compared to public-sector: Quota System in examination t(422)=-3.951,p=0.000, stress caused by lack of time for friends and family t(422)=-3.225,p=0.001, stress caused by Tests/Examination t(422)=-2.131,p=0.034, stress caused by the parental wish for them to study medicine t(422)=-2.346,p=0.019 and stress caused by fear of getting poor marks t(422)=-2.183,p=0.030.

**Conclusions:**

There exists no overall difference in the perceived-stress among the medical students of public and private medical colleges despite private-sectors having significantly more operational financial resources.

**Disclosure:**

No significant relationships.

